# Predictive value of CHA2DS2-VASc score combined with hs-CRP for new-onset atrial fibrillation in elderly patients with acute myocardial infarction

**DOI:** 10.1186/s12872-021-01978-8

**Published:** 2021-04-13

**Authors:** Yuan Fu, Yuxia Pan, Yuanfeng Gao, Xinchun Yang, Mulei Chen

**Affiliations:** Department of Cardiology, Chaoyang Hospital, Capital Medical University, Beijing, China

**Keywords:** New-onset atrial fibrillation, Acute myocardial infarction, CHA2DS2-VASc score, Elderly, Risk estimation

## Abstract

**Background:**

New-onset atrial fibrillation (NOAF) is common during acute myocardial infarction (AMI) and independently associated with worse prognosis. We aimed to validate the discrimination performance of CHA2DS2-VASc score combined with hs-CRP in the prediction of NOAF after AMI in elderly Chinese population.

**Methods:**

311 consecutive elderly patients (age ≥ 65 years old) with AMI from 1 January 2018 to 1 January 2019 without atrial fibrillation history were enrolled in our study. Univariable and multivariable logistic regression analyses were used to identify risk factors of NOAF. The discrimination performance of different score models were evaluated using ROC curve analysis and AUCs were compared using the Z test.

**Results:**

30 (9.65%) patients developed NOAF during hospitalization. The NOAF group were older and had higher hs-CRP, initial Killip class, BNP, LAD, CHADS2 score, CHA2DS2-VASc score, in-hospital mortality and lower LVEF and ACEI/ARB use (*P* < 0.05 vs group without NOAF for all measures). In multivariate regression analyses, age (OR = 1.127, 95% CI 1.063–1.196, *P* < 0.001) and hs-CRP (OR = 1.034, 95% CI 1.018–1.05, *P* < 0.001) were independent predictors of NOAF. In ROC curve analyses, both CHADS2 score (AUC = 0.624, 95% CI 0.516–0.733, *P* = 0.026) and CHA2DS2-VASc score (AUC = 0.687, 95% CI 0.584–0.79, *P* = 0.001) had acceptable but unsatisfactory discrimination performance in predicting NOAF after AMI. The combined model with CHA2DS2-VASc score and hs-CRP showed a significant better predictive value (AUC = 0.791, 95% CI 0.692–0.891, *P* < 0.001) compared to that of the CHA2DS2-VASc score alone (Z test, *P* = 0.008).

**Conclusion:**

The combined model with CHA2DS2-VASc score and hs-CRP had high accuracy in predicting post-AMI NOAF.

## Introduction

New-onset atrial fibrillation (NOAF) is a common arrhythmia during acute myocardial infarction (AMI), with a incidence ranging from 3 to 20% [1]. The development of NOAF in AMI patients has been proved as an independent factor of short- and long-term mortality [1, 2] Therefore, early identification of patients with high risk of NOAF is essential to prevent complications and improve prognosis. Previous studies have demonstrated several risk factors associated with NOAF, such as older age, B-type natriuretic peptide (BNP), high sensitive c-reactive protein (hs-CRP) and left atrium diameter (LAD) [3–6]. However, valid risk stratification model to predict NOAF during AMI remains unclear, especially in elderly AMI population.

The CHADS2 and the CHA2DS2-VASc score have been demonstrated as good predictors of stroke risk in patients with non-valvular atrial fibrillation (NVAF) [7, 8]. However, the discriminate power seems unsatisfactory when applying these two score models in AMI population for the prediction of NOAF [9, 10]. The aim of our study was to identify risk factors of NOAF in elderly patients hospitalized for AMI, and determine whether the CHA2DS2-VASc score combined with admission biomarkers, such as hs-CRP, can be a valid model to predict NOAF.

## Methods

### Study population

This is a one-center retrospective study of 314 consecutive elderly patients (age ≥ 65 years old) with AMI who admitted to Beijing Chaoyang Hospital between 1 January 2018 and 1 January 2019, without a history of pre-existing atrial fibrillation (AF). 3 patients with incomplete medical record were excluded from the present study. Patients were divided into two groups: with NOAF and without NOAF during hospitalization. This study was approved by the Ethics Committee of Beijing Chaoyang Hospital (2020-3-17-16). Since this is a retrospective analysis, informed consent was not applicable.

AMI was classified as ST-segment elevation myocardial infarction (STEMI) and non-ST-segment elevation myocardial infarction (NSTEMI). The Diagnostic criteria of STEMI were: (1) typical, prolonged ischemic symptoms (usually > 30 min) consistent with coronary artery disease (CAD); (2) a typical rise and fall of serum cardiac troponin-I (CTnI); (3) ST-segment elevation ≥ 1 mm in 2 or more contiguous leads on a 12-lead electrocardiogram (ECG), or a newly developed left bundle branch block (LBBB) on an initial ECG [11]. NSTEMI was defined as: (1) typical, prolonged ischemic symptoms (usually > 30 min) consistent with CAD and a typical rise and fall of serum CTnI; (2) absence of ECG changes of STEMI criteria, for example, with ST-segment depression and/or prominent T-wave inversion on an initial ECG [12]. Patients with type 2 myocardial infarction were excluded in the present study [13].

An initial ECG was conducted in the first 5 min after patients’ admission and all patients received continuous electrocardiography monitoring to detect arrhythmia during AMI. AF was diagnosed as the absence of P waves, an irregular R-R interval, an unidentifiable isoelectric line and lasting at least 30 s. [8] NOAF was defined as patients with no pre-existing AF who presented with sinus rhythm on admission and developed AF during hospital stay. NOAF was classified as early-NOAF (onset within 24 h after admission) and late-NOAF (onset after 24 h after admission) [14].

### Data collection

#### Anthropometric measurements and general data collection

Data about patients’ demographics, family and medical history, weight, height and status of smoking were collected upon their admission. BMI was calculated as weight divided by height squared (kg/m^2^). Estimated glomerular filtration rate (eGFR) was calculated with Modification of Diet in Renal Disease (MDRD) formula (Chinese version) [15]. Transthoracic echocardiography was performed within the first 6 h after patients’ admission. Left atrial diameter (LAD) was measured by M mode ultrasound at the parasternal view. The left ventricular end-systolic diameter (LVESd), left ventricular end-diastolic diameter (LVEDd) and left ventricular ejection fraction (LVEF) were evaluated using the Simpson’s method. Coronary angiography (CAG) information was collected and infarct related artery (IRA) was identified by CAG results combined with ECG changes.

### Risk score calculation

The CHADS2 score was calculated by assigning 1 point each for congestive heart failure (CHF), hypertension, age ≥ 75 years and diabetes mellitus (DM), and 2 points for previous stroke or transient ischemic attack (TIA) [16]. The CHA2DS2-VASc score was calculated by assigning 1 point each for CHF, hypertension, age 65–74 years, DM, vascular disease and female gender, and 2 points for age ≥ 75 years and previous stroke or TIA [17].

### Laboratory parameters

Venous blood samples (5 mL) were collected from the antecubital vein immediately after an initial ECG recording, and then analyzed with a Dimension RxLMax™ automated analyzer. All biochemical variables were measured with Hitachi 7600 automatic analyzer.

### Statistical analysis

Continuous variables were tested for normal distribution using the Kolmogorov–Smirnov test. Normally-distributed data are presented as mean ± standard deviation (SD) and analyzed by the Student’s t-test. Non-normally distributed variables are presented as median (interquartile range) and analyzed by the Mann–Whitney U test. Dichotomous variables were presented as percentages and were analyzed with the Pearson Chi-squared test. Univariable analysis and multivariable logistic regression were used to identify the risk factors of NOAF. Receiver operating characteristic (ROC) curve and the area under the curve (AUC) were analyzed to evaluate the discrimination performance of the CHADS2 and the CHA2DS2-VASc score. Youden index equals to sensitivity + specificity-1 [18]. AUC = 1.0 represents perfect discriminatory ability while AUC < 0.5 indicates the absence of predictive power [19]. The predictive power (AUCs) of different risk scores were compared using Z test. A 2-tailed *P* < 0.05 was considered statistically significant. SPSS 24.0 (IBM Corp, Armonk, NY) and STATA software (Version 16.0; Stata Corporation) were used for all statistical analyses.

## Results

### General characteristics

A total of 311 consecutive elderly AMI patients (81.03% male) with no history of AF were enrolled in the present study, and the mean age of all participants was 75.26 ± 9.84 years old. Baseline characteristics of relevant patients are shown in Table 1. The median follow-up time was 11 (6–16) days. Thirty patients (9.65%) developed NOAF during hospitalization: 8 (2.57%) early-NOAF cases and 22 (7.07%) late-NOAF cases. The median time of NOAF onset was 42 (20.5, 72) hours, with the earliest NOAF happened 4 h after admission and the latest NOAF happened 168 h after admission. NOAF patients were older and more likely to have higher Killip class, hs-CRP, B-type natriuretic peptide (BNP), LAD, the CHADS2 score, the CHA2DS2-VASc score and in-hospital mortality (*P* < 0.05 vs. patients without NOAF for all measures). Meanwhile, LVEF and angiotensin-converting enzyme inhibitor (ACEI)/ angiotensin receptor blocker (ARB) use were lower in NOAF group (*P* < 0.05 vs. patients without NOAF for all measures).

**Table 1:** Baseline characteristics of the study population

Variables	NOAF (n = 30)	Without NOAF (n = 281)	*P* value
Age, years	82.43 ± 9.21	73.37 ± 10.31	< 0.001
Male, n (%)	23 (76.67)	229 (81.49)	0.322
STEMI, n (%)	19 (63.33)	175 (62.78)	0.925
HT, n (%)	18 (60)	156 (55.52)	0.761
DM, n (%)	9 (30)	95 (33.81)	0.546
History of MI, n (%)	8 (26.67)	50 (17.79)	0.179
History of PCI, n (%)	5 (16.67)	39 (13.88)	0.159
History of CABG, n (%)	2 (6.67)	7 (2.49)	0.192
History of CHF, n (%)	2 (6.67)	29 (10.32)	0.564
History of stroke, n (%)	6 (20)	38 (13.52)	0.218
Current smoker, n (%)	16 (53.33)	166 (59.07)	0.45
BMI, kg/m^2^	24.33 ± 2.39	26.19 ± 3.07	0.061
SBP, mmHg	122.37 ± 22.82	126.49 ± 19.83	0.172
DBP, mmHg	66.37 ± 12.41	65.49 ± 13.29	0.223
HR at admission, bpm	74 ± 13.6	78.56 ± 12.49	0.074
IRA			
RCA, n (%)	8 (26.67)	92 (32.74)	0.304
LAD, n (%)	13 (43.33)	123 (43.77)	0.932
LCX, n (%)	7 (23.33)	57 (20.28)	0.21
Killip class			
I, n (%)	7 (23.33)	118 (41.99)	0.003
II, n (%)	13 (43.33)	132 (46.98)	0.317
III, n (%)	8 (26.67)	22 (7.83)	0.001
IV, n (%)	2 (6.67)	9 (3.2)	0.135
WBC, 10^9^/L	9.88 ± 2.83	10.34 ± 3.42	0.543
Hb, g/L	135.78 ± 20.36	139.74 ± 19.18	0.263
PLT, 10^9^/L	210.2 ± 58.74	223.17 ± 65.39	0.053
HbAlc, %	6.1 (5.85–7.4)	6.3 (5.6–7.2)	0.684
ESR, mm/h	11.3 (5.55–24.78)	8.1 (4.08–15.1)	0.084
Hs-CRP, mg/L	28.03 (2.64–91.38)	5.2 (2.21–19.04)	0.003
D-dimer, mg/L FEU	0.35 (0.22–0.89)	0.29 (0.2–0.61)	0.144
CK-MB, ng/ml	18.54 (9.6–100.44)	35.29 (6.2–133.41)	0.677
CTnI, ng/ml	17.22 (7.11–106.3)	30.01 (6.32–109.71)	0.747
TC, mmol/L	4.3 ± 1.02	4.49 ± 1.11	0.169
TG, mmol/L	1.37 (0.92–1.77)	1.41 (1.01–2.1)	0.171
LDL-C, mmol/L	2.67 ± 1.18	3.03 ± 1.16	0.079
HDL-C, mmol/L	0.97 ± 0.24	0.96 ± 0.25	0.994
LP(a), mg/dl	16.4 (8.51–23.6)	14.04 (8.43–29.82)	0.961
BNP, pg/ml	384 (184–886.41)	151.3 (72.56–278.53)	< 0.001
SCR, µmol/L	68.1 (56.1–95.47)	67.42 (58.77–81.39)	0.948
eGFR, ml/min/1.73 m^2^	99.37 ± 32.51	107.24 ± 30.57	0.125
SUA, umol/L	397.81 ± 141.27	384.19 ± 103.99	0.211
LVEF, %	53.6 (40.72–63.19)	61.4 (48.79–66.3)	0.023
LVESd, mm	34 (30.1–38.1)	32 (28–39)	0.109
LVEDd, mm	48.4 (44.91–54.2)	48 (44–52)	0.362
LAD,mm	38.1 (34.7–42.59)	35.2 (33.2–38.4)	0.031
Medications			
ACEI/ARB, n (%)	5 (16.67)	107 (38.08)	0.046
Beta blocker, n (%)	14 (46.67)	186 (66.19)	0.069
Statin, n (%)	28 (93.33)	272 (96.78)	0.367
IABP, n (%)	5 (16.67)	28 (9.96)	0.055
CHADS2 score	2 (1–3)	1 (1–2)	0.019
CHA2DS2-VASc score	4 (3–5)	3 (2–4)	0.001
In-hospital mortality, n (%)	3 (10)	3 (1.06)	< 0.001

Data are number (%), mean (SD), or median (IQR)

NOAF, new-onset atrial fibrillation; STEMI, ST-segment elevation myocardial infarction; HT, hypertension; DM, diabetes mellitus; MI, myocardial infarction; PCI, percutaneous coronary intervention; CABG, coronary artery bypass grafting; CHF, chronic heart failure; BMI, body mass Index; SBP, systolic blood pressure; DBP, diastolic blood pressure; HR, heart rate; IRA, infarct related artery; RCA, right coronary artery; LAD, left anterior descending artery; LCX, left circumflex coronary artery; WBC, white blood cell; Hb, haemoglobin; PLT, platelet; HbAlc, glycosylated hemoglobin; ESR, erythrocyte sedimentation rate; Hs-CRP, high sensitive c-reactive protein; HCY, homocysteine; CK-MB, creatine kinase MB; CTnI, cardiac troponin I; TC, total cholesterol; TG, triglyceride; LDL-C, low-density lipoprotein cholesterol; HDL-C, high-density lipoprotein cholesterol; LP(a), Lipoprotein (a); BNP, B-type natriuretic peptide; SCR, serum creatinine; eGFR, estimated glomerular filtration rate; SUA, serum uric acid; LVEF, left ventricular ejection fraction; LVESd, left ventricular end-systolic diameter; LVEDd, left ventricular end-diastolic diameter; LAD, left atrium diameter; ACEI, angiotensin-converting enzyme inhibitor; ARB, angiotensin receptor blocker; IABP, intra-aortic ballon pump

### The discriminatory ability of CHADS2 and CHA2DS2-VASc score

**Fig. 1:**
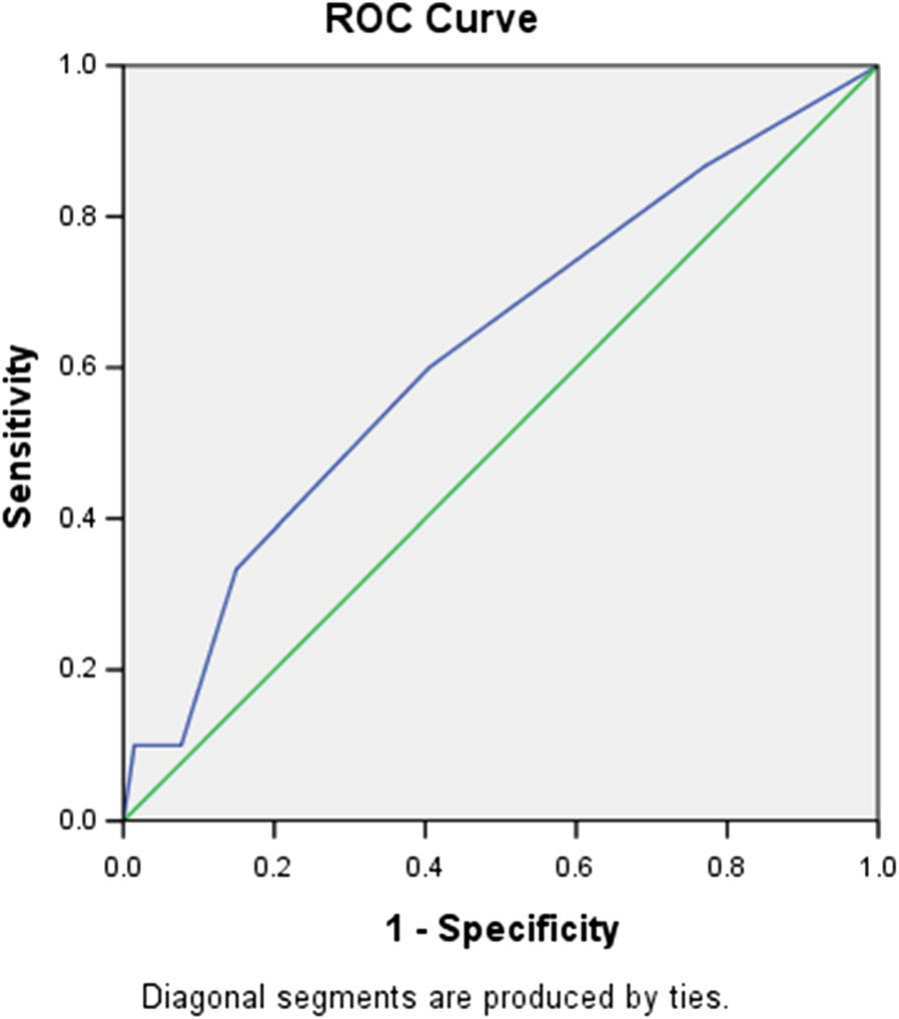
Receiver operating characteristic curve analysis for the CHADS2 score in predicting post-AMI NOAF. The area under the curve was 0.624 (95% CI 0.516–0.733, *P* = 0.026)

**Fig. 2:**
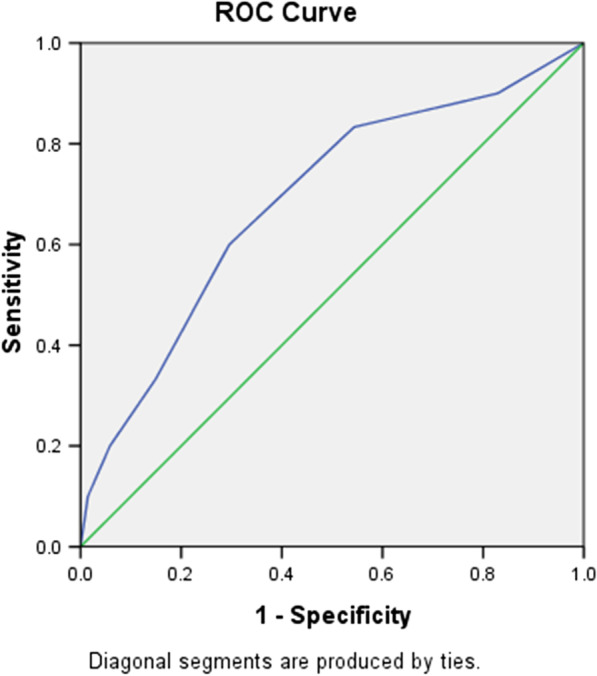
Receiver operating characteristic curve analysis for the CHA2DS2-VASc score in predicting post-AMI NOAF. The area under the curve was 0.687 (95% CI 0.584–0.79, *P* = 0.001)

The CHADS2 and the CHA2DS2-VASc score of NOAF patients were significantly higher than that of patients without NOAF [2 (1–3) vs. 1 (1–2), *P* = 0.019; 4 (3–5) vs. 3 (2–4), *P* = 0.001; Table 1]. ROC curve analyses were performed and both the CHADS2 score and the CHA2DS2-VASc score showed an acceptable discriminatory ability for the prediction of NOAF after AMI as evidenced by an AUC = 0.624 (95% CI 0.516–0.733, *P* = 0.026, Fig. 1) and an AUC = 0.687 (95% CI 0.584–0.79, *P* = 0.001, Fig. 2), respectively.

### Independent risk factors of NOAF

**Fig. 3:**
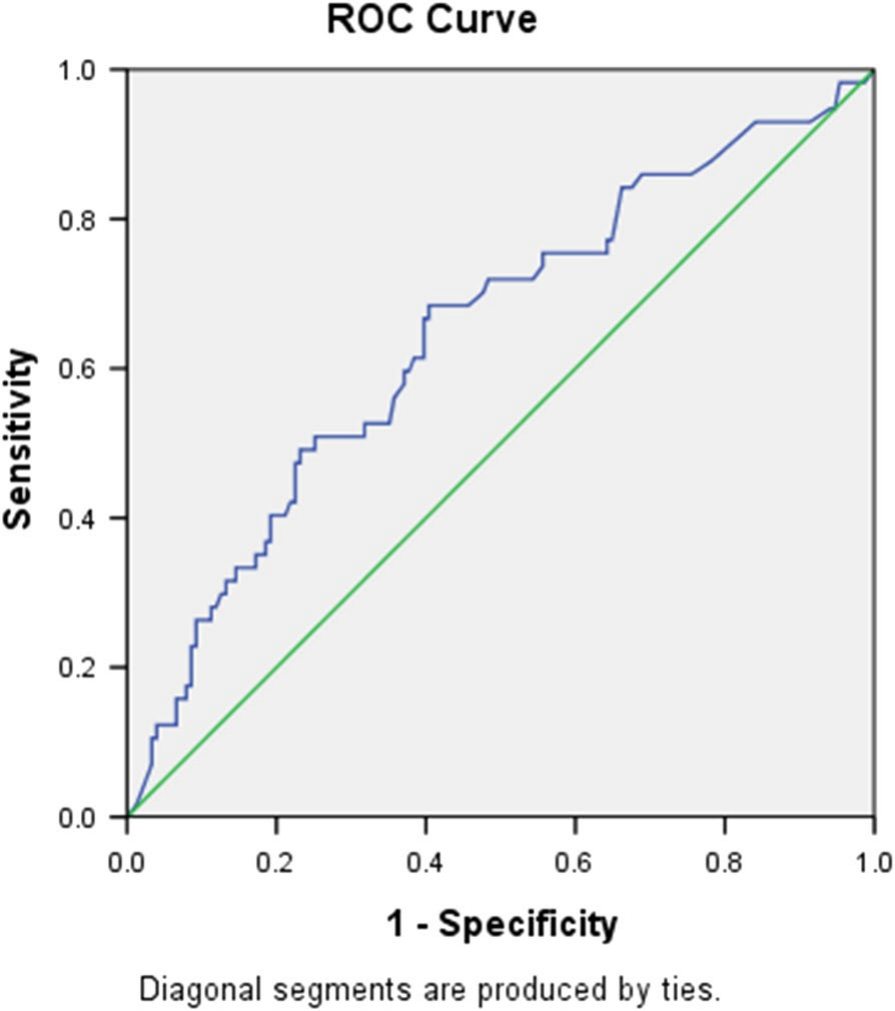
Receiver operating characteristic curve analysis for the hs-CRP in predicting post-AMI NOAF. The area under the curve was 0.671 (95% CI 0.535–0.807, *P* = 0.004)

In multivariate logistic regression analyses, age and hs-CRP remained as independent predictors of NOAF after the adjustment for clinical characteristics (BNP, Killip class and ACEI/ARB use, Table 2, model 1) and echocardiography index (LAD and LVEF, Table 2, model 2). Logistic regression analyses that included the CHADS2 and the CHA2DS2-VASc score as independent variables also indicated that hs-CRP is an independent risk factor of NOAF (Table 2, model 3 and 4). The ROC curve analysis demonstrated that the best cut-off point of hs-CRP to predict post-AMI NOAF was 21.25 mg/L (sensitivity:57.69%, specificity: 81.74%, AUC 0.671, 95% CI 0.535–0.807, *P* = 0.004, Fig. 3).

**Table 2:** Multiple logistic regression analyses for independent risk factors of NOAF

	Estimated β	OR (95% CI)	*P* value
Model 1			
**Age, years**	**0.12**	**1.127 (1.063–1.196)**	***P < 0.001***
**Hs-CRP, mg/L**	**0.034**	**1.034 (1.018–1.05)**	***P < 0.001***
BNP, pg/ml	0.001	1 (0.999–1.001)	0.446
Model 2			
**Age, years**	**0.118**	**1.125 (1.036–1.221)**	**0.005**
**Hs-CRP, mg/L**	**0.026**	**1.026 (1.01–1.048)**	**0.013**
LAD, mm	0.055	1.057 (0.921–1.213)	0.43
LVEF, %	− 0.018	0.983 (0.932–1.036)	0.516
Model 3			
**Hs-CRP, mg/L**	**0.028**	**1.028 (1.016–1.04)**	***P < 0.001***
**CHADS2 score**	**0.338**	**1.403 (1.025–1.92)**	**0.035**
Model 4			
**Hs-CRP, mg/L**	**0.028**	**1.028 (1.015–1.041)**	***P < 0.001***
**CHA2DS2-VASc score**	**0.425**	**1.529 (1.173–1.994)**	**0.002**

Variables included in model 1 are age, hs-CRP and BNP. Variables included in model 2 are age, hs-CRP, LAD and LVEF. Variables included in model 3 are hs-CRP and the CHADS2 score. Variables included in model 4 are hs-CRP and the CHA2DS2-VASc score

NOAF, new-onset atrial fibrillation; Hs-CRP, high sensitive c-reactive protein; BNP, B-type natriuretic peptide; ACEI, angiotensin-converting enzyme inhibitor; ARB, angiotensin receptor blocker; LAD, left atrium diameter; LVEF, left ventricular ejection fraction;

### ROC curve analysis: the CHA2DS2-VASc score combined with hs-CRP

**Fig. 4:**
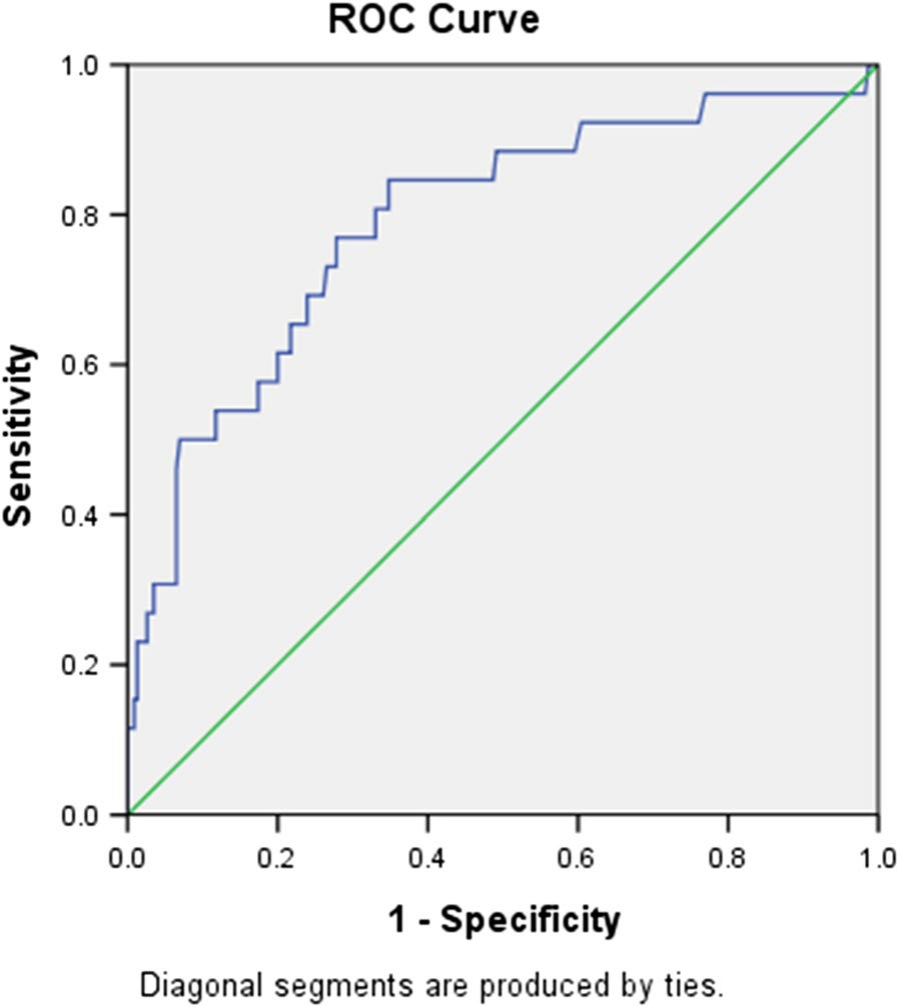
Receiver operating characteristic curve analysis for the CHA2DS2-VASc score combined with hs-CRP in predicting post-AMI NOAF. The area under the curve was 0.791 (95% CI 0.692–0.891, *P* < 0.001)

**Fig. 5:**
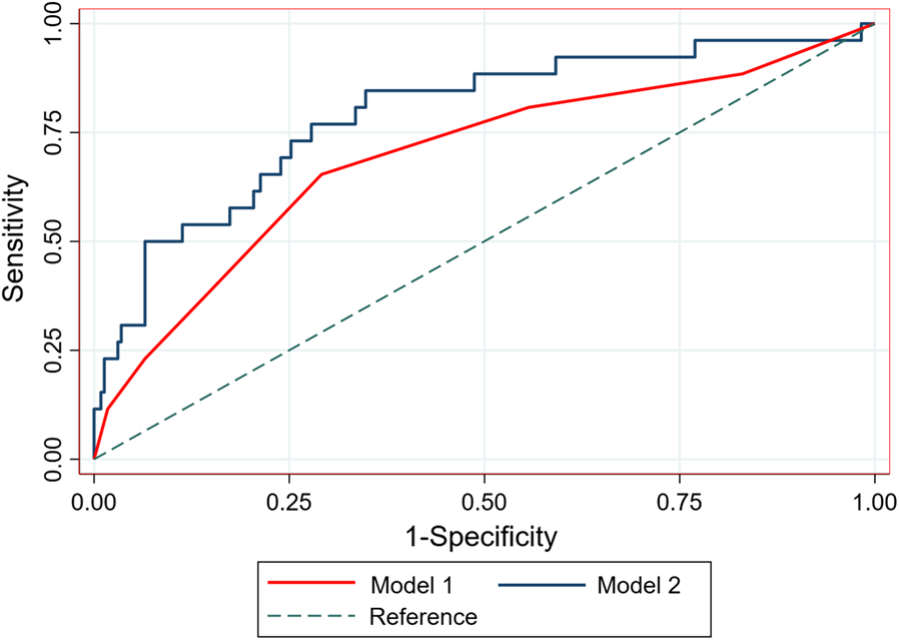
The comparison of areas under the curve using Z test. The AUC of model 2 was significantly higher than that of model 1 (*P* = 0.008). Abbreviations: Model 1 was the CHA2DS2-VASc score, and Model 2 was the CHA2DS2-VASc score combined with hs-CRP

Hs-CRP was proved as an independent risk factor of NOAF and we wanted to evaluate the additive prognostic value of it in addition to the CHA2DS2-VASc score. AS a result, another ROC curve analysis was performed and AUC of the CHA2DS2-VASc score combined with hs-CRP to predict NOAF after AMI in elderly population was 0.791 (95% CI 0.692–0.891, *P* < 0.001, Fig. 4), significantly higher than that of the CHA2DS2-VASc score alone. (Z test, 0.791 vs. 0.687, *P* = 0.008, Fig. 5).

## Discussion

The main findings of the present study were: (1) older age and high hs-CRP were two independent risk factors of NOAF in elderly Chinese AMI patients. (2) the CHA2DS2-VASc score system is a convenient model to predict post-AMI NOAF with acceptable but unsatisfactory discriminatory ability. The predictive value of the score can be significantly enhanced by combing with hs-CRP.

Occurrence of NOAF during the course of AMI is a common phenomenon and it is associated with worse prognosis such as increased thromboembolic events, heart failure (HF) and all-cause mortality [1, 2, 20]. The incidence of post-AMI NOAF was 9.65% in our study, which is consistent with the incidence reported in previous studies (3–20%) [1, 14]. Moreover, the in-hospital mortality was significant higher in NOAF group (10% vs. 1.06%, *P* < 0.001). Most NOAF (7.07%) developed after 24 h after admission (late-NOAF) with the ratio of late-NOAF to early-NOAF was nearly 3 to 1, similar results were reported in former surveys [14, 21].

Several risk factors of post-AMI NOAF have been reported in previous studies, such as older age, higher Killip class, hs-CRP, BNP, LAD and lower LVEF [3, 5, 6, 22]. For instance, a study by Wang et al. showed that older age is independently related to NOAF in patients with ACS, and similar conclusions were showed in some other studies [3, 5, 23]. In the present study, older age was still an independent risk factor of post-AMI NOAF, even we only focused on elderly population. A study by Parashar et al. demonstrated that admission biomarkers such as hs-CRP and NT-proBNP were independently associated with NOAF, irrespective of gender and race [6]. We observed a similar results in our study as well. However, after multivariate logistic regression analyses, only one admission blood biomarker, hs-CRP, was independent risk factor of NOAF in elderly AMI population.

The mechanisms for the association between increased hs-CRP and NOAF is not entirely clear. Previous studies suggest that inflammation is involved in the development and persistence of AF [9, 24]. Hs-CRP is a simple biomarker for the magnitude of inflammation and higher hs-CRP level observed in NOAF group may suggest a potential link between the more severe systemic inflammation caused by myocardial necrosis and the development of NOAF after AMI [25]. Moreover, CRP can bind to the phosphocholine groups exposed in the membrane of necrotic cardiomyocyte, causing the activation of complement and thus leading to more intense CRP deposition and local inflammation, which may facilitate the genesis and development of post-AMI NOAF [26]. In our study, when elderly AMI patients present with hs-CRP > 21.25 mg/L at admission, they are more likely to develop NOAF during hospitalization.

The CHADS2 and the CHA2DS2-VASc score are used for thromboembolic risk stratification and have been recommended by modern guideline in the management of patients with NVAF [27]. However, the discriminative value of these two score models in predicting post-AMI NOAF was unsatisfactory [9, 10]. In the study by Lau et al., when applying the CHADS2 and the CHA2DS2-VASc score for the prediction of post-AMI NOAF, the discriminate power was relatively poor as evidenced by a AUC of 0.632 and 0.676, respectively [10]. In our study, the CHADS2 and the CHA2DS2-VASc score were significantly higher in NOAF group, and we observed a similar discriminate power evidenced by AUC (0.624 and 0.687, respectively). These evidences also illustrating the need for a scoring model that is more accurate than the CHA2DS2-VASc score in predicting post-AMI NOAF.

For this purpose, we combined the CHA2DS2-VASc score with hs-CRP. The AUC of the combined model was statistically greater than that of the CHA2DS2-VASc score (0.791 vs. 0.687, *P* = 0.008) after Z test, suggesting the diagnostic performance of this new model to predict NOAF in elderly AMI patients was relatively high and significantly better than the CHA2DS2-VASc score alone. With the use of this new combined model, it will be convenient to identify elderly patients with AMI who are at higher risk to develop NOAF, and help clinicians in making therapeutic strategies to improve prognosis. For instance, previous studies had proved the protective value of statins use against AF in ACS patients, thus aggressive statin therapy might be beneficial [28]. Moreover, AF patients benefit from anticoagulant therapy, early identification of patients with higher risk of NOAF and early diagnosis of NOAF has clinical importance for a timely initiation and adjustment of anticoagulant therapy [27]. However, these beneficial effects should be verified in future randomized controlled trials.

## Limitations

As a single-center retrospective study, the sample size was relatively small, which could limit the number of risk factor identified. The potential cause-effect relationship could not be determined as well. Thus, the present findings should be warranted in future large multicenter trials.

## Conclusions

Older age and high hs-CRP at admission were independent predictors of NOAF in elderly Chinese patients presenting with AMI. The discriminate power of the CHA2DS2-VASc score in predicting post-AMI NOAF was acceptable, and could be enhanced significantly when combing with hs-CRP.

## Data Availability

The datasets generated and/or analysed during the current study are not publicly available due to the restrictions by the Beijing Chaoyang Hospital, but are available from the corresponding author on reasonable request.

## Abbreviations

NOAF: New-onset atrial fibrillation
AMI: Acute myocardial infarction
AF: Atrial fibrillation
BNP: B-type natriuretic peptide
LAD: Left atrium diameter
LVEF: Left ventricular ejection fraction
ACEI: Angiotensin-converting enzyme inhibitor
ARB: Angiotensin receptor blocker
Hs-CRP: High sensitive c-reactive protein
CRP: C-reactive protein
NVAF: Non-valvular atrial fibrillation
STEMI: ST-segment elevation myocardial infarction
NSTEMI: Non-ST-segment elevation myocardial infarction
CAD: Coronary artery disease
CTnI: Cardiac troponin-I
LBBB: Left bundle branch block
ECG: Electrocardiogram
eGFR: Estimated glomerular filtration rate
MDRD: Modification of Diet in Renal Disease
LVESd: Left ventricular end-systolic diameter
LVEDd: Left ventricular end-diastolic diameter
CAG: Coronary angiography
IRA: Infarct related artery
CHF: Congestive heart failure
DM: Diabetes mellitus
TIA: Transient ischemic attack
SD: Standard deviation
ROC: Receiver operating characteristic
AUC: Area under the curve

## References

[1] Jabre P, Roger VL, Murad MH, Chamberlain AM, Prokop L, Adnet F, Jouven X (2011). Mortality associated with atrial fibrillation in patients with myocardial infarction: a systematic review and meta-analysis. Circulation.

[2] Lopes RD, Pieper KS, Horton JR, Al-Khatib SM, Newby LK, Mehta RH, Van de Werf F, Armstrong PW, Mahaffey KW, Harrington RA, Ohman EM, White HD, Wallentin L, Granger CB (2008). Short- and long-term outcomes following atrial fibrillation in patients with acute coronary syndromes with or without ST-segment elevation. Heart.

[3] He J, Yang Y, Zhang G, Lu XH (2019). Clinical risk factors for new-onset atrial fibrillation in acute myocardial infarction: a systematic review and meta-analysis. Medicine.

[4] Karabag Y, Rencuzogullari I, Cagdas M, Karakoyun S, Yesin M, Uluganyan M, Gursoy MO, Artac I, Ilis D, Gokdeniz T, Efe SC, Tasar O, Tanboga HI (2018). Association between BNP levels and new-onset atrial fibrillation: a propensity score approach. Herz.

[5] Wang J, Yang YM, Zhu J (2015). Mechanisms of new-onset atrial fibrillation complicating acute coronary syndrome. Herz.

[6] Parashar S, Kella D, Reid KJ, Spertus JA, Tang F, Langberg J, Vaccarino V, Kontos MC, Lopes RD, Lloyd MS (2013). New-onset atrial fibrillation after acute myocardial infarction and its relation to admission biomarkers (from the TRIUMPH registry). Am J Cardiol.

[7] Malik R, Alyeshmerni DM, Wang Z, Goldstein SA, Torguson R, Lindsay J, Waksman R, Ben-Dor I (2015). Prevalence and predictors of left atrial thrombus in patients with atrial fibrillation: is transesophageal echocardiography necessary before cardioversion?. Cardiovasc Revascularization Med Incl Mol Interv.

[8] Kirchhof P, Benussi S, Kotecha D, Ahlsson A, Atar D, Casadei B, Castella M, Diener HC, Heidbuchel H, Hendriks J, Hindricks G, Manolis AS, Oldgren J, Popescu BA, Schotten U, Van Putte B, Vardas P, Agewall S, Camm J, Baron Esquivias G, Budts W, Carerj S, Casselman F, Coca A, De Caterina R, Deftereos S, Dobrev D, Ferro JM, Filippatos G, Fitzsimons D, Gorenek B, Guenoun M, Hohnloser SH, Kolh P, Lip GY, Manolis A, McMurray J, Ponikowski P, Rosenhek R, Ruschitzka F, Savelieva I, Sharma S, Suwalski P, Tamargo JL, Taylor CJ, Van Gelder IC, Voors AA, Windecker S, Zamorano JL, Zeppenfeld K (2016). 2016 ESC Guidelines for the management of atrial fibrillation developed in collaboration with EACTS. Europace Eur Pacing Arrhythm Card Electrophysiol J Work Groups Card Pacing Arrhythm Card Cell Electrophysiol Eur Soc Cardiol.

[9] Huang SS, Chan WL, Leu HB, Huang PH, Chen JW, Lin SJ (2013). Association between CHADS2 score and the preventive effect of statin therapy on new-onset atrial fibrillation in patients with acute myocardial infarction. PLoS ONE.

[10] Lau KK, Chan PH, Yiu KH, Chan YH, Liu S, Chan KH, Yeung CY, Li SW, Tse HF, Siu CW (2014). Roles of the CHADS2 and CHA2DS2-VASc scores in post-myocardial infarction patients: risk of new occurrence of atrial fibrillation and ischemic stroke. Cardiol J.

[11] Ibanez B, James S, Agewall S, Antunes MJ, Bucciarelli-Ducci C, Bueno H, Caforio ALP, Crea F, Goudevenos JA, Halvorsen S, Hindricks G, Kastrati A, Lenzen MJ, Prescott E, Roffi M, Valgimigli M, Varenhorst C, Vranckx P, Widimsky P, Group ESCSD (2018). 2017 ESC Guidelines for the management of acute myocardial infarction in patients presenting with ST-segment elevation: The Task Force for the management of acute myocardial infarction in patients presenting with ST-segment elevation of the European Society of Cardiology (ESC). Eur Heart J.

[12] Roffi M, Patrono C, Collet JP, Mueller C, Valgimigli M, Andreotti F, Bax JJ, Borger MA, Brotons C, Chew DP, Gencer B, Hasenfuss G, Kjeldsen K, Lancellotti P, Landmesser U, Mehilli J, Mukherjee D, Storey RF, Windecker S, Group ESCSD (2016). 2015 ESC Guidelines for the management of acute coronary syndromes in patients presenting without persistent ST-segment elevation: Task Force for the Management of Acute Coronary Syndromes in Patients Presenting without Persistent ST-Segment Elevation of the European Society of Cardiology (ESC). Eur Heart J.

[13] Hung J, Roos A, Kadesjo E, McAllister DA, Kimenai DM, Shah ASV, Anand A, Strachan FE, Fox KAA, Mills NL, Chapman AR, Holzmann MJ (2020). Performance of the GRACE 2.0 score in patients with type 1 and type 2 myocardial infarction. Eur Heart J.

[14] Shiyovich A, Axelrod M, Gilutz H, Plakht Y (2019). Early versus late new-onset atrial fibrillation in acute myocardial infarction: differences in clinical characteristics and predictors. Angiology.

[15] Ma YC, Zuo L, Chen JH, Luo Q, Yu XQ, Li Y, Xu JS, Huang SM, Wang LN, Huang W, Wang M, Xu GB, Wang HY (2006). Modified glomerular filtration rate estimating equation for Chinese patients with chronic kidney disease. J Am Soc Nephrol.

[16] Olesen JB, Lip GYH, Hansen ML, Hansen PR, Tolstrup JS, Lindhardsen J, Selmer C, Ahlehoff O, Olsen AMS, Gislason GH, Torp-Pedersen C (2011). Validation of risk stratification schemes for predicting stroke and thromboembolism in patients with atrial fibrillation: nationwide cohort study. BMJ.

[17] January C, Wann L, Alpert J, Calkins H, Cigarroa J, Cleveland JJ, Conti J, Ellinor P, Ezekowitz M, Field M, Murray K, Sacco R, Stevenson W, Tchou P, Tracy C, Yancy C, Guidelines. ACoCAHATFoP (2014). 2014 AHA/ACC/HRS guideline for the management of patients with atrial fibrillation: a report of the American College of Cardiology/American Heart Association Task Force on Practice Guidelines and the Heart Rhythm Society. J Am Coll Cardiol.

[18] Bantis LE, Nakas CT, Reiser B (2014). Construction of confidence regions in the ROC space after the estimation of the optimal Youden index-based cut-off point. Biometrics.

[19] Meulendijks D, van Hasselt JGC, Huitema ADR, van Tinteren H, Deenen MJ, Beijnen JH, Cats A, Schellens JHM (2016). Renal function, body surface area, and age are associated with risk of early-onset fluoropyrimidine-associated toxicity in patients treated with capecitabine-based anticancer regimens in daily clinical care. Eur J Cancer.

[20] Kundu A, O'Day K, Shaikh AY, Lessard DM, Saczynski JS, Yarzebski J, Darling CE, Thabet R, Akhter MW, Floyd KC, Goldberg RJ, McManus DD (2016). Relation of atrial fibrillation in acute myocardial infarction to in-hospital complications and early hospital readmission. Am J Cardiol.

[21] McManus DD, Huang W, Domakonda KV, Ward J, Saczysnki JS, Gore JM, Goldberg RJ (2012). Trends in atrial fibrillation in patients hospitalized with an acute coronary syndrome. Am J Med.

[22] Ulus T, Isgandarov K, Yilmaz AS, Vasi I, Moghanchizadeh SH, Mutlu F (2018). Predictors of new-onset atrial fibrillation in elderly patients with acute coronary syndrome undergoing percutaneous coronary intervention. Aging Clin Exp Res.

[23] Schmitt J, Duray G, Gersh BJ, Hohnloser SH (2009). Atrial fibrillation in acute myocardial infarction: a systematic review of the incidence, clinical features and prognostic implications. Eur Heart J.

[24] Engelmann MD, Svendsen JH (2005). Inflammation in the genesis and perpetuation of atrial fibrillation. Eur Heart J.

[25] Aronson D, Boulos M, Suleiman A, Bidoosi S, Agmon Y, Kapeliovich M, Beyar R, Markiewicz W, Hammerman H, Suleiman M (2007). Relation of C-reactive protein and new-onset atrial fibrillation in patients with acute myocardial infarction. Am J Cardiol.

[26] Lagrand WK, Visser CA, Hermens WT, Niessen HW, Verheugt FW, Wolbink GJ, Hack CE (1999). C-reactive protein as a cardiovascular risk factor: more than an epiphenomenon?. Circulation.

[27] Hindricks G, Potpara T, Dagres N, Arbelo E, Bax JJ, Blomstrom-Lundqvist C, Boriani G, Castella M, Dan GA, Dilaveris PE, Fauchier L, Filippatos G, Kalman JM, La Meir M, Lane DA, Lebeau JP, Lettino M, Lip GYH, Pinto FJ, Thomas GN, Valgimigli M, Van Gelder IC, Van Putte BP, Watkins CL, Group ESCSD (2020). 2020 ESC Guidelines for the diagnosis and management of atrial fibrillation developed in collaboration with the European Association of Cardio-Thoracic Surgery (EACTS). Eur Heart J.

[28] Ozaydin M, Turker Y, Erdogan D, Karabacak M, Dogan A, Varol E, Gonul E, Altinbas A (2010). The association between previous statin use and development of atrial fibrillation in patients presenting with acute coronary syndrome. Int J Cardiol.

